# Exosomes-mediated transfer of LINC00691 regulates the formation of CAFs and promotes the progression of gastric cancer

**DOI:** 10.1186/s12885-023-11373-5

**Published:** 2023-10-02

**Authors:** Bin Xia, Xiuyu Gu, Tingting Xu, Meina Yan, Lan Huang, Chun Jiang, Meifen Li, Guanghua Zhai, Guoping Zhang, Jian Wu, Yu Zhou, Chunrong Sun, Wei Liang

**Affiliations:** 1 Department of Laboratory Medicine, Suzhou Hospital, Affiliated Hospital of Medical School, Nanjing University, Suzhou, 215153 China; 2 Department of Laboratory Medicine, Gusu School, The Affiliated Suzhou Hospital of Nanjing Medical University, Suzhou Municipal Hospital, Nanjing Medical University, Suzhou, 215008 China; 3 Department of General Surgery, Gusu School, The Affiliated Suzhou Hospital of Nanjing Medical University, Suzhou Municipal Hospital, Nanjing Medical University, Suzhou, 215008 China

**Keywords:** Exosomes, Cancer-associated fibroblasts, Normal fibroblasts, lncRNAs, Gastric cancer

## Abstract

**Objective:**

Gastric cancer (GC) is one of the malignant tumors with the highest mortality worldwide. Our previous studies have revealed that LINC00691 is up-regulated in serum of GC patients as a novel potential biomarker for GC diagnosis and prognosis. However, the roles of serum exosomal LINC00691 in GC has not been clarified. This study aimed to find the expression pattern of serum exosomal LINC00691 in GC patients and the correlation between the level of serum exosomal LINC00691 and the pathology of gastric cancer patients.

**Methods:**

We collected the serum of 94 GC patients before surgery and extracted exosomes to detect the expression level of exosomal LINC00691, with 21 healthy volunteers and 17 patients with benign gastric diseases as controls. Surgical GC tissues and paired healthy tissues were collected to culture primary cancer-associated fibroblasts (CAFs) and normal fibroblasts (NFs). We then treated NFs with LINC00691-rich GC cell culture supernatant or exosomes and detected the activation markers and biological functions of the fibroblasts.

**Results:**

The results of real-time qPCR indicated that the serum exosomal LINC00691 of GC patients was significantly higher than that of healthy subjects and patients with benign gastric diseases, and was associated with the clinicopathology of GC patients. More interestingly, when the NFs were treated with GC exosomes, the level of LINC00691 was significantly increased, the cell proliferation and migration were noticeably enhanced, and the ability to accelerate GC cell proliferation and invasion was promoted, which means that the induced fibroblasts gained the properties of CAFs. In addition, we found that knockdown of LINC00691 and the use of the JAK2/STAT3 signaling pathway inhibitor ruxolitinib effectively deprived exosome-containing GC cell supernatants of the effects on NFs.

**Conclusion:**

Our study suggested that exosomal LINC00691 promoted NFs to gained the properties of CAFs depending on JAK2/STAT3 signaling pathway as a potential diagnostic biomarker for GC.

**Supplementary Information:**

The online version contains supplementary material available at 10.1186/s12885-023-11373-5.

GC is the fifth most common malignant tumor and the third leading cause of cancer-related mortality worldwide, causing nearly 800,000 deaths every year [1, 2]. Early diagnosis of GC is a crucial prerequisite for effective clinical intervention. Pathological tissue biopsy is the gold method for definite diagnosis of GC although it is not appropriate to take it as a screening method in population because of invasive damage. Peripheral blood biomarkers, especially CEA, CA72-4, and CA19-9, are classical clinical screening means for GC [3]. However, the area under the curve (AUC) of the receiver operating characteristic curve (ROC) is respectively 0.653, 0.685, and 0.639, which is not satisfactory clinically [4]. Therefore, it is badly in need to search for new ideal biomarkers for GC diagnosis and prognosis [5, 6].

Systematic analysis studies have shown that the non-coding genome accounts for approximately 98% of all sequences, the aberrant expression of which is the main cause raising the transformation of tumor phenotype [7]. Long non-coding RNAs (lncRNAs) mainly refer to RNAs with more than 200 nucleotides and do not code proteins [8]. However, in recent years, a series of lncRNAs have been proved to produce small peptides [9]. In our previous study, we found that LINC00691 level was associated with tumor size, lymph node metastasis, invasion depth, and prognosis of patients. LINC00691 was overexpressed in GC and the ROC curve was better than traditional classical indicators [10]. In this study, we found the AUC of serum exosomal LINC00691 even larger than serum LINC00691.

Exosomes are vesicles secreted by cells that can carry biologically active substances [11, 12]. Studies have shown that exosomes from tumor cells regulate cell reprogramming, thereby promoting the transformation of NFs to CAFs. Moreover, this effect may rely on the Janus kinase/signal transducer and activator of transcription (JAK/STAT) signaling pathway [13]. We previously reported that LINC00691 promoted GC cell proliferation and invasion through the JAK2/STAT3 signaling pathway [10]. The JAK/STAT signaling pathway has been reported as a common pathway in the regulation of many cellular biological activities [14, 15]. Notably, the JAK/STAT signaling pathway has been reported to be very important in hematological diseases [16] and many solid tumors, such as cervical cancer [17], breast cancer [18], hepatocellular carcinoma [19, 20], and acute myeloid leukemia (AML) [21]. Therefore, we proposed the hypothesis that exosomes might promote the transformation of NFs into CAFs depending on LINC00691 via JAK / STAT signaling pathway.

Our previous research results showed that exosomes contained in GC cell culture supernatants carry lncRNAs into cells and cause transformation in biological function [22]. The tumor microenvironment is composed of microenvironmental cells and an extracellular matrix, which plays crucial regulatory roles in almost all kinds of tumors [23]. CAFs are the vital stromal components that promote tumor progression in various ways [24]. In addition, the normal stroma of non-tumor tissues also contains a number of normal fibroblasts cells, which may be educated into CAFs. The high expression of α-smooth muscle actin (α-SMA) in CAFs is an important sign of activated fibroblasts [25].

This study aimed to estimate the clinical value of exosomal LINC00691 in the diagnosis assessment of GC and explore the mechanism of promoting CAFs formation through the JAK2/STAT3 signaling pathway.

## Materials and methods

### Patients and serum samples

A total of 94 serum samples were obtained from GC patients who were diagnosed with primary GC confirmed by pathological diagnosis in Nanjing Medical University Affiliated Suzhou Hospital from April 2019 to March 2022. All patients had not received surgical treatment, radiation therapy, chemotherapy or other adjuvant therapy before enrollment and had no other primary malignant tumors of digestive system or other systems. We also collected serum samples from 17 patients with benign gastric diseases including gastritis, gastric ulcer, and benign tumors diagnosed by endoscopy and pathology. In addition, 21 serum samples were obtained from healthy volunteers as control subjects. We have gotten informed consent from all of the mentioned patients and volunteers above. Also, this study was carried out after approval by the Institutional Ethical Committee of Nanjing Medical University on 2022 − 02–23. The number of the Ethics Committee decision is No. (2022)169.

### Cell lines and cell culture

Human GC cell line HGC-27 and MKN-45(Guangzhou Cellcook Biotech Co., Ltd., Guangzhou, China) were cultured in complete medium, which contains 90% RPMI 1640 medium (Invitrogen, Carlsbad, CA) and 10% fetal bovine serum (FBS) in a CO_2_ incubator at 37 °C. Primary NFs and CAFs were collected from tumor tissues and paired normal samples. Briefly, the tissue was washed 3 times with PBS, soaked in 10,000 U of penicillin and streptomycin for 15 min, and cut into small pieces of 4 mm ^2^. And then 20% FBS-containing fibroblast medium (ScienCell Research Laboratories, San Diego, CA) was added for culture until the fibroblasts around the tissue block were confluent, and then they were digested by trypsin and passaged. In this study, we used NFs and CAFs of passages 5–8.

### Exosome extraction

To exclude the effect of exosomes in serum, we centrifuged at 100,000 g for 18 h before extracting exosomes in the cell culture supernatant. The cell culture supernatant was collected and filtered using an exosome enrichment column (Merck Millipore Ltd. Darmstadt, Germany). Exosomes were then extracted using exosome extraction reagents (System Bioscience, LLC Palo Alto, CA) according to the protocol provided by the manufacturer.

### RNA extraction and quantitative real-time polymerase chain reaction (qRT-PCR)

Total RNA was extracted via TRIzol reagent (Invitrogen) and then a HiFiScript cDNA Synthesis Kit (Cwbio, Beijing, China) was used to produce cDNA. Subsequently, RealSYBR Mixture (Cwbio) was applied in real-time qPCR performed in a real-time PCR Detection System (LightCycler 480, Roche, Switzerland). U6 was used as an internal reference. The sequences of primers used in this study were as follows: LINC00691-F: 5’-GCTCCCGATAAGCAACTGGA-3’; LINC00691-R: 5’-ACTCGTAGCCCAGAATCCCT-3’; U6-F: 5’-CTCGCTTCGGCAGCACA-3’; U6-R: 5’- AACGCTTCACGAATTTGCGT-3’.

### Transmission electron microscopy (TEM)

Transmission electron microscopy was applied for exosome morphology. Briefly, 1% glutaraldehyde was used to fix exosomes in PBS and then a drop (20 μl) of exosomes was placed on 200-mesh Formvar/carbon-coated nickel grids, which were stained with 3% (W/V) aqueous phospphotungstic aid. The morphology of exosomes was recorded by transmission electron microscopy (Tecnai 12; Philips).

### Nanoparticle size analysis

The exosome solution was diluted 10,000 times before being loaded on the nanoparticle size analyzer (#NanoSight NS300, Malven, England). It was carried out in accordance with the operating procedures provided by the manufacturer.

### Immunofluorescence

Cells were fixed with 4% paraformaldehyde for 15 min and washed three times with PBS for 5 min each. Subsequently, cells were blocked with 0.3% Triton™ X-100 buffer for 60 min at room temperature. After blocking, the primary antibody was added and incubated overnight at 4 °C. Then, rinse three times with PBS for 5 min each before adding secondary antibody and incubating specimens for 1 h in the dark. Before photographing with a fluorescence microscope, three washes with PBS were performed for 5 min each.

### Treatment of supernatant of cell culture medium

After fresh cell culture supernatant was collected, cell debris was removed by centrifugation at 1500 g for 10 min. When the cells were treated, an equal volume of the complete medium of the treated cells was added, and the cells were cultured at 37 °C with a 5% CO_2_ atmosphere for 48 h.

### Cell transfection

For relevant materials and procedures of cell transfection, please refer to our previous article [10].

Mainly, GC cells were seeded into a 6-well cell culture plate (2 × 10^5^ per well) and cultured overnight in 5% CO_2_ with antibiotic-free complete medium. LipoFiter transfection reagent (6 μl per well, Hanbio, Shanghai, China) was used to achieve the transfection of shRNAs targeting LINC00691 (1 μg per well, Hanbio) into GC cells in 1 ml serum-free RPMI-1640. The medium was then changed to complete medium containing 10% fetal bovine serum after transfection for 6 h. Subsequent experiments were performed at 36 h after transfection.

### Cell counting assay and cell colony formation assay

After a 36 h transfection, 5 × 10 ^4^ cells were seeded in 5 wells equally in 24-well plates. The total cell number was then counted every 24 h. GraphPad 5.0 was applied to draw growth curves depending on cell number. Also, cells were seeded in 6-well plates for colony formation assay (1 × 10^4^ per well) and cultured t 37 °C with a 5% CO_2_ atmosphere for 7 days. Subsequently, cells were fixed using 4% paraformaldehyde and then stained with crystal violet. The colony number was finally calculated using a microscope.

### Transwell migration and invasion assay

After a 36 h transfection, 2 × 10^4^ cells were seeded into the top chamber of Transwell chambers (Chemicon, Temecula, CA, USA) resuspended in a 200 μl serum-free medium. Complete medium (600 μl) was added into the bottom chamber. For invasion assay, there was an increased step compared to the migration experiment: the top chamber was added with 20% Matrigel matrix 8 h before seeding cells and the number of seeded cells was 1 × 10^5^. After migration or invasion for 36 h, cells were fixed using 4% paraformaldehyde for 30 min at room temperature, stained with crystal violet for 15 min, and counted under the microscope after the upper layer cells were carefully removed.

### Wound healing assay

A total of 2 × 10^4^ cells were seeded in a 24 well plate overnight, and then a straight line was drawn in the middle of every well with a 10 μl pipette tip. After being washed three times with PBS, cells were changed to a serum-free medium. Cells were then photographed and recorded after 0, 4, and 8 h in a CO_2_ incubator at 37 °C.

### Western blot analysis

RIPA buffer (Beyotime, Shanghai, China) was mixed with 0.1% protease inhibitor PMSF (Beyotime) and applied to lyse cells. The protein samples were denatured by heating and mixed with loading buffer before electrophoresis. SDS–polyacrylamide gel electrophoresis (SDS-PAGE) was performed to separate proteins. Then target proteins were electrophoretically transferred at 360 mA current for 120 min and collected on 0.22-μm polyvinylidene fluoride (PVDF) membranes (Millipore, MA, USA). 5% skim milk was used to block non-specific binding sites for 60 min at room temperature. PVDF membranes were then cropped prepared for applying different primary antibody. Primary antibody was applied to bind the target protein on PVDF membranes overnight at 4 °C. The membrane was washed three times for 5 min each with TBS/T and incubated with the secondary antibody for 60 min at 37 °C. Scientific SuperSignal chemiluminescent HRP substrates (Thermo Fisher, Waltham, USA) was then applied matched with an enhanced chemiluminescence kit according to the operating procedures provided by the manufacturer. GAPDH was applied as an internal reference.

### Statistical analysis

Mainly, we used SPSS 22.0 (Chicago, IL, USA) for statistical analyses and GraphPad Prime 5 (GraphPad, San Diego, CA) for statistical charts. All experiments mentioned in this study were repeated thrice at least. Values recorded in this study are performed as mean ± SD. Student’s t-test was applied to analyze the statistical difference between paired groups. ANOVA and Newman-Keuls Multiple Comparison Test was used to test whether there was any statistical difference among the three groups of treated samples. Pearson χ2 test was used to analyze if there was any correlation between serum exosomal LINC00691 level and clinicopathological features of GC patients. *P* < 0.05 was considered as a statistically significant difference.

## Results

### Exosomal LINC00691 level in serum of GC patients was higher than that of healthy people

**Fig. 1:**
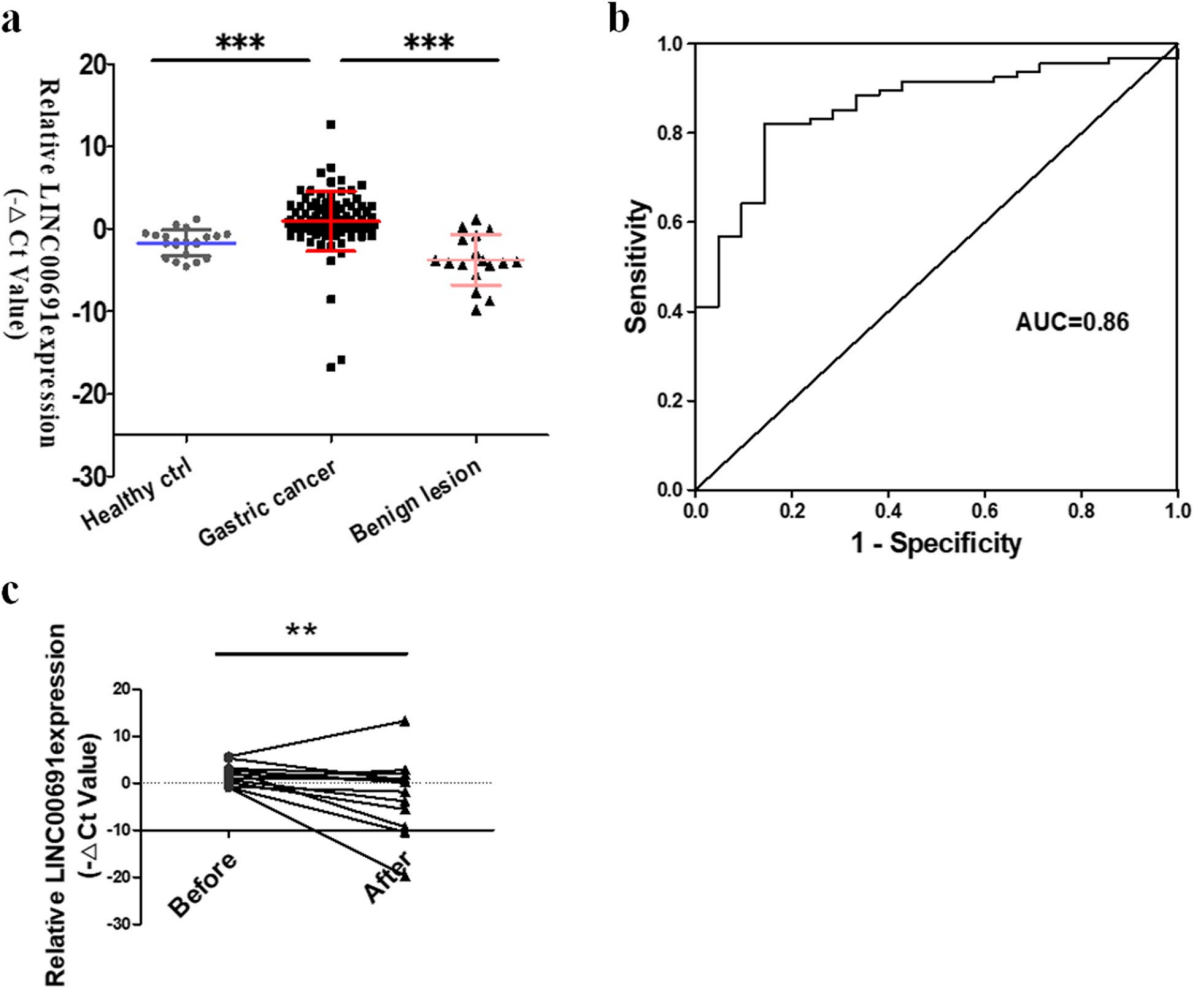
Exosomal LINC00691 level in serum of GC patients, patients with benign gastric diseases, and healthy people.** a** Scatter plot of serum exosomal LINC00691 levels in patients with GC (*n* = 94), benign gastric diseases (*n* = 17), and healthy people (*n* = 21). The expression levels of Exosomal LINC00691 were determined by using qRT-PCR (***P < 0.001). Data were presented as mean ± SD. **b** The ROC curve of serum exosomal LINC00691 in GC. **c** Changes of serum exosomal LINC00691 levels in GC patients before and after GC resection (**P < 0.01)

We extracted serum exosomes from 94 GC patients and detected the expression of LINC00691. The results indicated that the LINC00691 level in serum exosomes of GC patients was significantly higher than that in healthy people and patients with gastric benign disease (Fig. 1a). Moreover, the results of pathological correlation analysis showed that the level of serum exosomal LINC00691 was associated with tumor size, lymph node metastasis, vascular invasion and perineural invasion of patients (Table 1). The area under the curve (AUC) was 0.86, which was higher than that of serum LINC00691. The sensitivity was 0.85 and the specificity was 0.71 respectively (Fig. 1b). These results demonstrated that LINC00691 in serum exosomes may be an ideal biomarker for GC. Besides, we collected a part of GC patients' serum after gastrectomy and detected the expression of exosomal LINC00691. The results manifested that the exosomal LINC00691 level in the serum of GC patients was significantly reduced after gastrectomy (Fig. 1c).

**Table 1:** The association between serum exosomal LINC00691 expression levels (–ΔCt) and the clinicopathological features of gastric cancer patients

Features	Number	LINC00691		Mean ± SD	*P* value	χ^2^
		**Low**	**High**			
**Gender**						
Male	68	34	34	0.96 ± 3.06	1.000	< 0.001
Female	26	13	13	0.72 ± 4.94		
**Age (years)**						
< 60	28	14	14	1.40 ± 3.63	1.000	< 0.001
≥ 60	66	33	33	0.68 ± 3.67		
**Tumor size(cm)**						
< 3	57	40	17	-0.08 ± 3.04	< 0.001	21.572
≥ 3	37	7	30	2.39 ± 4.03		
**Differentiation**						
Poor	68	32	36	0.93 ± 3.31	0.490	0.479
Moderate	26	15	11	0.80 ± 4.50		
**Lymphatic metastasis**						
Absent	48	32	16	0.29 ± 3.78	0.002	9.579
Present	46	15	31	1.52 ± 3.44		
**Venous invasion**						
Absent	60	37	23	0.42 ± 3.58	0.005	7.787
Present	34	10	24	1.74 ± 3.67		
**Perineural invasion**						
Absent	65	40	25	0.56 ± 3.52	0.002	9.774
Present	29	7	22	1.65 ± 3.88		
**Invasion depth**						
T0	43	25	18	0.22 ± 3.52	0.214	1.543
T1	51	22	29	1.46 ± 3.70		
**Histopathological typing**						
Adenocarcinoma	79	37	42	0.83 ± 3.66	0.084	4.959
Signet-ring cell carcinoma	8	7	1	1.00 ± 4.90		
Others	7	3	4	1.46 ± 2.03		
**Tumor location**						
Cardia	37	21	16	0.48 ± 3.37	0.608	1.839
Antrum	23	11	12	0.38 ± 2.82		
Body	25	10	15	1.77 ± 4.95		
Others	9	5	4	1.49 ± 2.03		

"T1" indicates that the tumor has infiltrated the entire layer, while "T0" indicates that the tumor has not infiltrated the entire layer

### Isolation and identification of primary NFs and CAFs

**Fig. 2:**
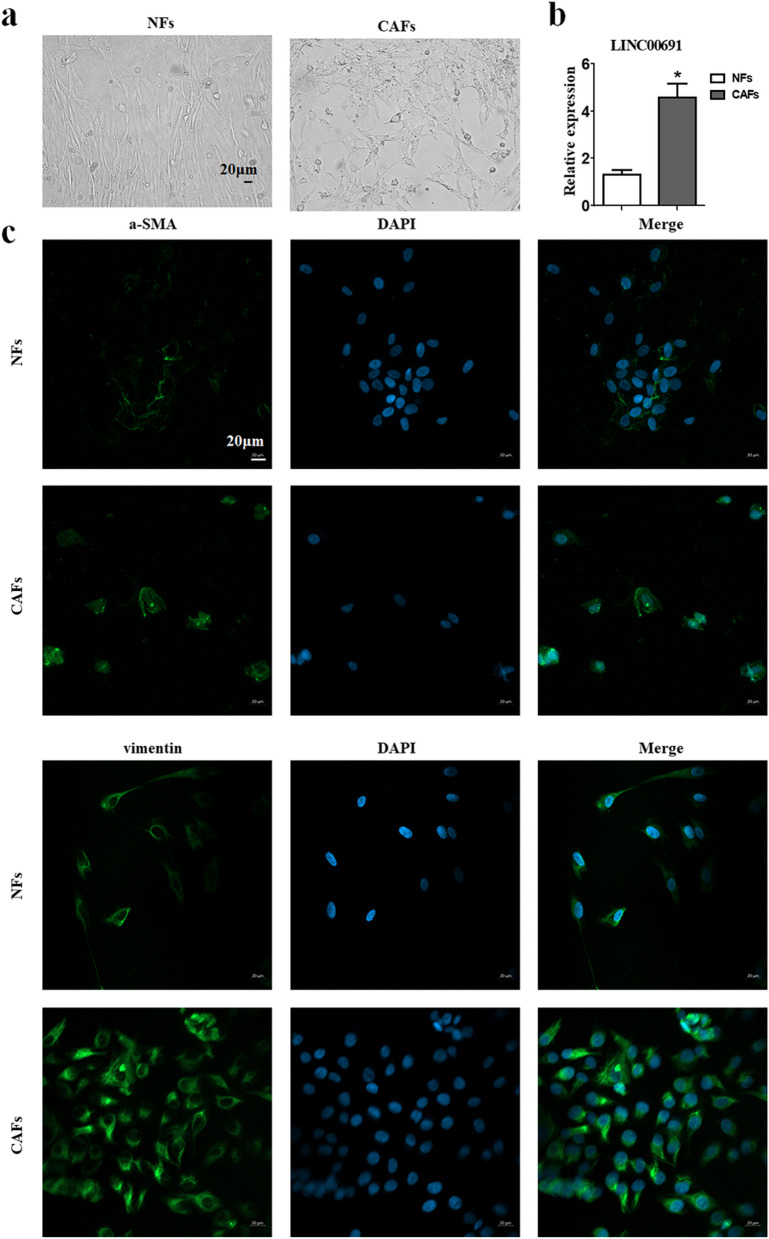
Isolation and identification of primary NFs and CAFs. **a** The morphology of NFs and CAFs under the microscope. Magnification, × 200. Scale bar, 20 μm. **b** The expression levels of LINC00691 in NFs and CAFs were determined by using qRT-PCR (*P < 0.05). **c** Immunofluorescence staining of α-SMA and Vimentin in NFs and CAFs. Magnification, × 200. Scale bar, 20 µm

We isolated and cultured CAFs and NFs from GC tumor tissues and paired healthy tissues. We observed microscopically similar morphologies of NFs and CAFs, but the former was a sinner and more elongated with relatively fewer cell processes (Fig. 2a). We collected CAFs and NFs in generations 3–8 and found the expression of LINC00691 in CAFs (4.58 ± 0.99) was higher than that in NFs (1.33 ± 0.33) (Fig. 2b). Since α-SMA and Vimentin have been reported as activation markers of CAFs, we examined their distribution in NFs and CAFs by immunofluorescence. The results indicated that the expression of both α-SMA and Vimentin in CAFs was higher than NFs (Fig. 2c).

### Treatment of NFs with exosomes from GC cells enhances the cell viability of NFs

**Fig. 3:**
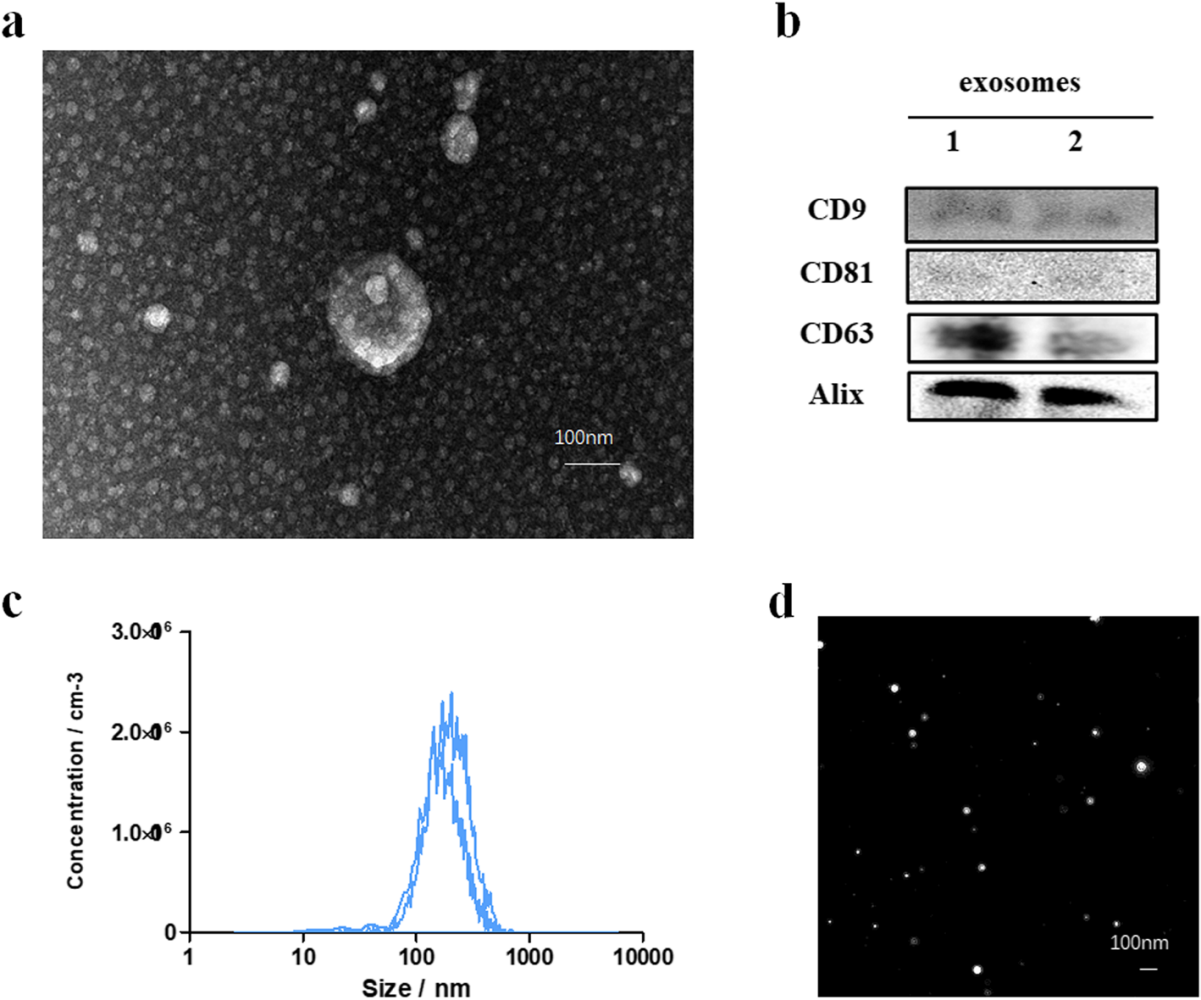
Identification of exosomes. **a** Morphology of exosomes photographed by transmission electron microscopy (TEM). Scale bar, 100 nm. **b** Western blot detection of exosome marker molecules CD9, CD81, CD63, and Alix. **c** Nanoparticle size analysis of the particle size/concentration of exosomes from HGC-27 cells. **d** Morphology of exosomes photographed by (Nanoparticle Tracking Analysis, NTA)

To explore the effects of exosomes from GC cells on NFs, we isolated and detected exosomes from GC cells. It was found that exosomes in GC cell supernatants were spherical vesicles of 40–150 nm in size using transmission electron microscopy (TEM) (Fig. 3a). Western blot results showed that purified exosomes expressed exosomal biomarkers CD9, CD81, CD63, and Alix (Fig. 3b). we also used NTA to analyze the size distribution and concentration of exosomes. Approximately, isolated exosomes were mainly distributed in diameters ranging from 50 to 200 nm in size and the concentration ranged from 1.0 × 10 ^11^ to 2.5 × 10 ^10^ particles/ml (Fig. 3c). Exosomes observed by NTA showed similar morphology to that under electron microscope (Fig. 3d).

**Fig. 4:**
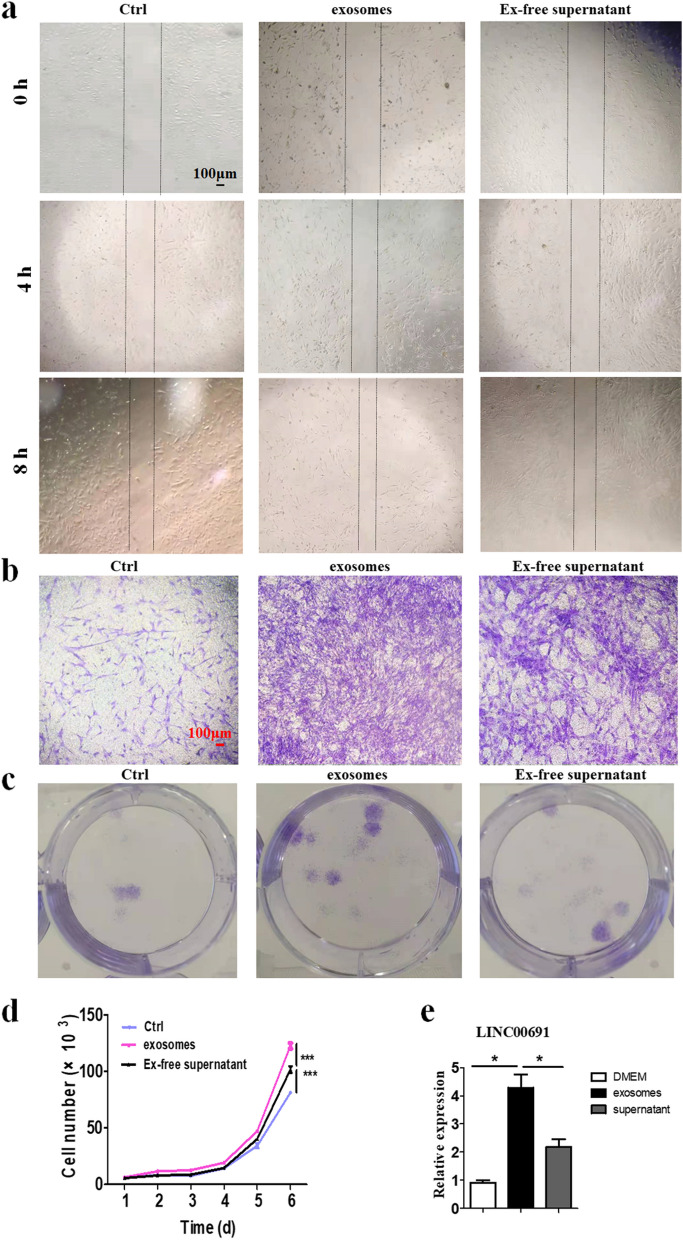
NFs treated with exosomes from GC cell culture supernatant.** a** Wound healing assay of fibroblasts after being treated with exosomes. Magnification, × 40. Scale bar, 100 µm. **b** Transwell migration assay of fibroblasts after being treated with exosomes. Magnification, × 40. Scale bar, 100 µm. **c** Cell colony formation assay of fibroblasts after being treated with exosomes. Magnification, × 100. Scale bar, 20 µm. **d** Cell counting assay of fibroblasts after treated with exosomes. **e** The expression levels of LINC00691of fibroblasts after being treated with exosomes (*P* < 0.05, compared to ctrl)

We treated NFs with exosomes from GC cell culture supernatant and found that the migration of NFs was enhanced through wound healing assay and Transwell migration assay, which was significantly inhibited when NFs were treated with exosome-depleted GC cell culture supernatants (Fig. 4a, b). Plate cloning experiments showed that exosomes derived from GC cell culture supernatant promoted NFs to acquire stronger clonogenic ability. Nevertheless, this effect was distinctly suppressed when exosomes were removed by centrifugation (Fig. 4c). Similarly, growth curve assay indicated that GC cell culture supernatant-derived exosomes promoted NFs to acquire stronger proliferative capacity (Fig. 4d). Besides, exosomes derived from GC cell culture supernatant enhanced the expression of LINC00691in NFs (*P* < 0.05, compared to ctrl, Fig. 4e).

### GC cell culture supernatant treatment replicated the effects of exosome treatment

**Fig. 5:**
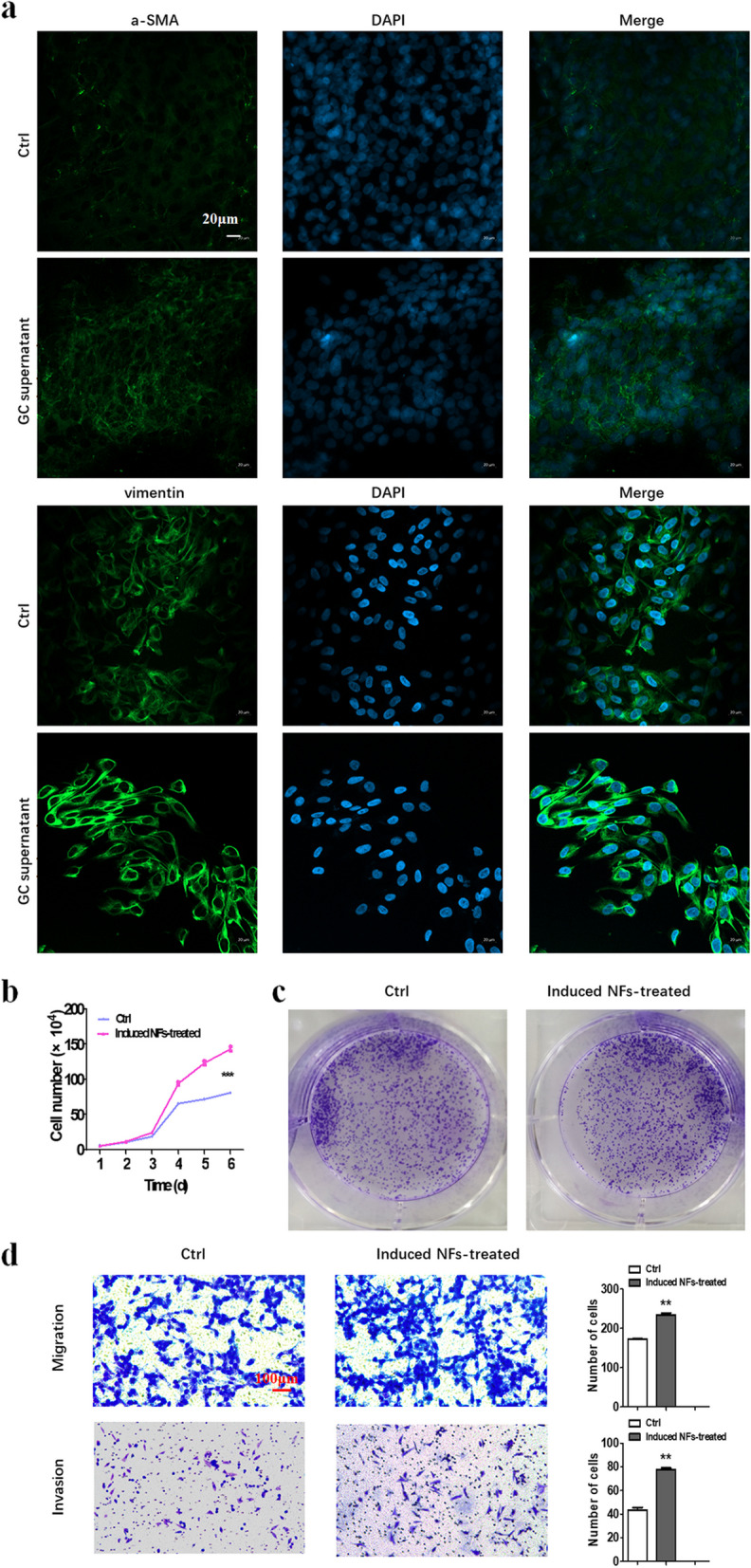
Biological effects of GC supernatants containing exosomes on NFs.** a** Immunofluorescence staining of α-SMA and Vimentin in fibroblasts after being treated with GC supernatants. Magnification, × 200. Scale bar, 20 µm. **b** Cell counting assay of GC cells after being treated with fibroblasts induced by supernatants. **c** Cell colony formation assay of GC cells after being treated with fibroblasts induced by supernatants. **d** Transwell migration and invasion assay of GC cells after being treated with fibroblasts induced by supernatants. Magnification, × 200. Scale bar, 10 µm, **P < 0.01

GC cell culture supernatant was applied to treat NFs and it was found that the expression of α-SMA and Vimentin in NFs was significantly increased (Fig. 5a). It has been reported that activated fibroblasts enhanced the proliferation and invasion of GC cells [1]. We subsequently treated GC cells with culture supernatant derived from the treated fibroblasts. The proliferation of GC cells was significantly enhanced when treated with fibroblasts pretreated with GC cell culture supernatants (Fig. 5b). Clonogenicity was likewise promoted by fibroblasts pretreated with GC cell culture supernatants (Fig. 5c). Transwell migration and invasion assay manifested that GC cells acquired reinforced migration and invasion abilities benefited from fibroblasts activated by GC cell culture supernatants (Fig. 5d).

### Knockdown of LINC00691 suppressed the effects of GC cell culture supernatant on NFs

**Fig. 6:**
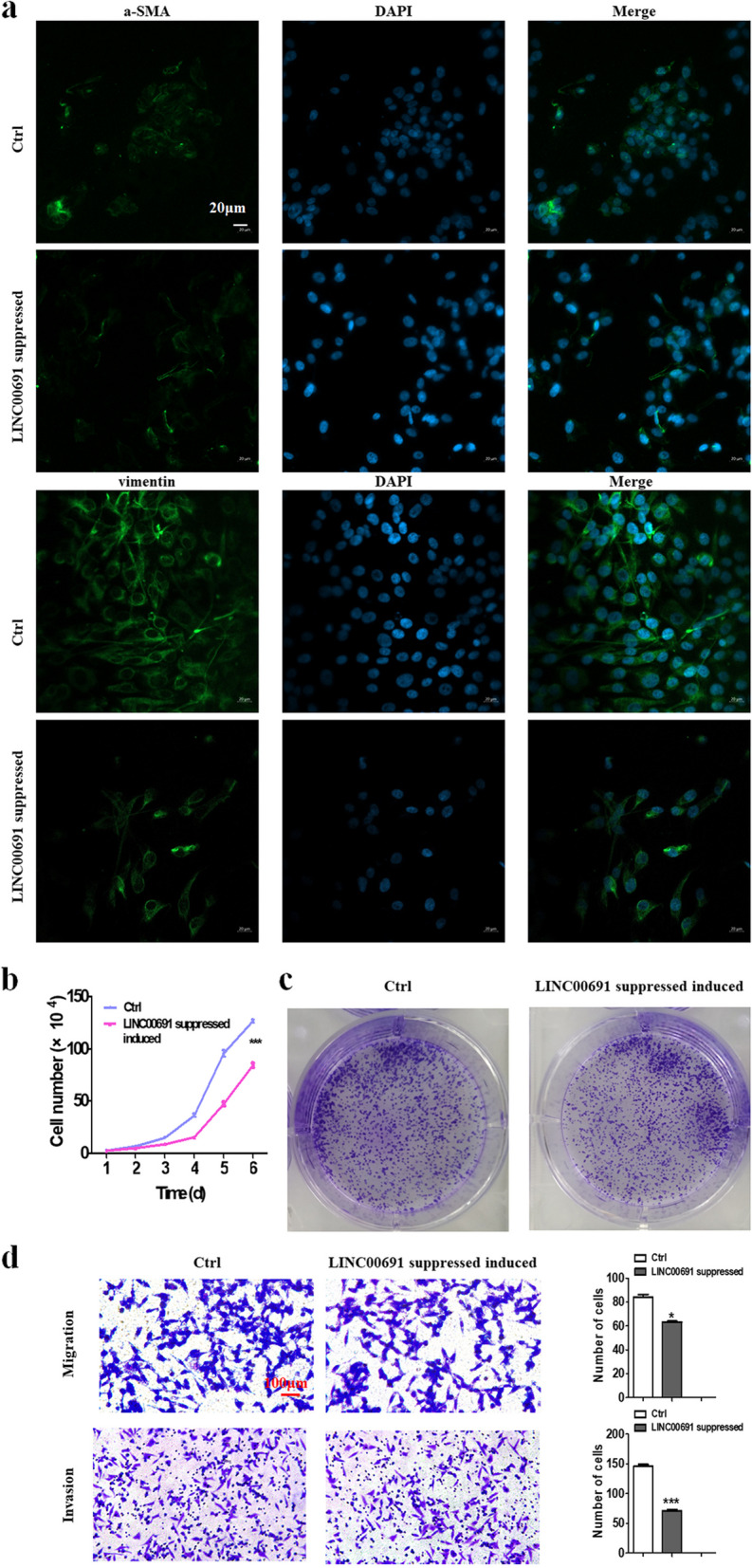
Biological effects of LINC00691 in GC supernatants on NFs. **a** Immunofluorescence staining of α-SMA and Vimentin in fibroblasts after being treated with LINC00691 knockdown GC supernatants. Magnification, × 200. Scale bar, 20 µm. **b** Cell counting assay of GC cells after being treated with fibroblasts induced by LINC00691 knockdown supernatants. **c** Cell colony formation assay of GC cells after being treated with fibroblasts induced by LINC00691 knockdown supernatants. **d** Transwell migration and invasion assay of GC cells after being treated with fibroblasts induced by LINC00691 knockdown supernatants. Magnification, × 200. Scale bar, 10 µm, *P < 0.05, ***P < 0.001

As we found the expression of LINC00691 of NFs was upregulated when treated with exosomes derived from GC cell culture supernatant (Fig. 4e), we then knockdown LINC00691 in GC cells and collected culture supernatant. The results indicated that the inhibition of LINC00691 led to the restraints of effects of GC cell culture supernatant on NFs. Specifically, the expression of fibroblast activated biomarkers α-SMA and Vimentin was significantly decreased (Fig. 6a). The proliferation, clonogenicity, migration, and invasion of GC cells were weakened when treated by pretreated fibroblasts compared with the control group (Fig. 6b-d).

### The inhibition of JAK2/STAT3 signaling pathway suppressed the effects of GC cell culture supernatant on NFs

**Fig. 7:**
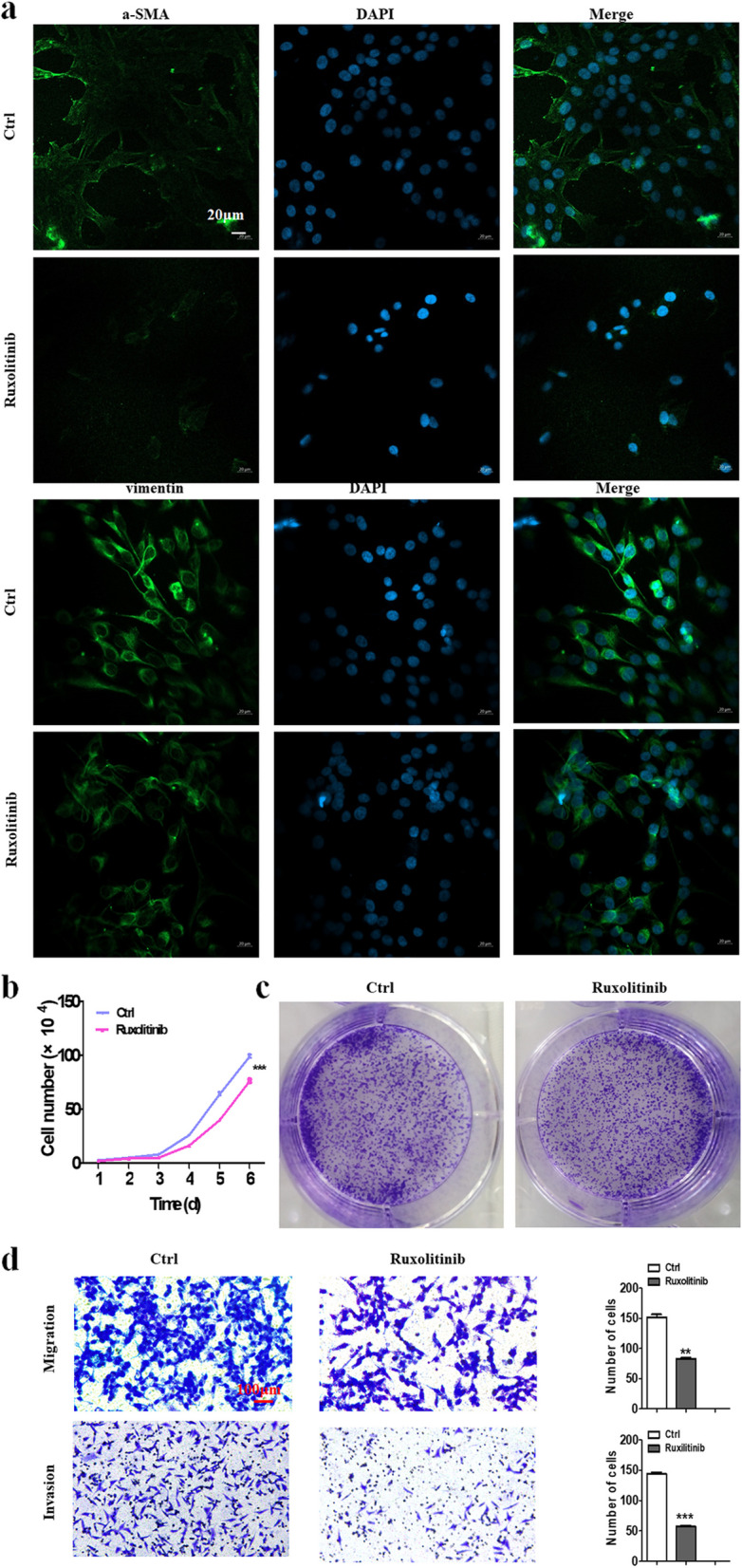
Biological effects of JAK2/STAT3 signaling pathway in GC supernatants on NFs. **a** Immunofluorescence staining of α-SMA and Vimentin in fibroblasts after being treated with ruxolitinib pre-treated GC supernatants. Magnification, × 200. Scale bar, 20 µm. **b** Cell counting assay of GC cells after being treated with fibroblasts induced by ruxolitinib pre-treated supernatants. **c** Cell colony formation assay of GC cells after being treated with fibroblasts induced by ruxolitinib pre-treated supernatants. **d** Transwell migration and invasion assay of GC cells after being treated with fibroblasts induced by ruxolitinib pre-treated supernatants. Magnification, × 200. Scale bar, 10 µm, **P < 0.01, ***P < 0.001

In previous studies, we elucidated that LINC00691 promoted GC malignant progression through the JAK2/STAT3 signaling pathway. Therefore, we use the inhibitor of the JAK2/STAT3 signaling pathway called ruxolitinib in the system when we applied GC cell culture supernatant to treat NFs. It was found that the expression of fibroblast activated biomarkers α-SMA and Vimentin was apparently reduced by ruxolitinib (Fig. 7a). Afterwards, we treated GC cells with culture supernatant derived from the treated fibroblasts. The results indicated that the effects of induced fibroblasts of promoting proliferation, clonogenicity, migration, and invasion on GC cells activated by GC cell culture supernatants were remarkably suppressed compared with the control group (Fig. 7b-d).

## Discussion

It is widely reported that lncRNAs play indispensable roles in various cell life process, including but not limited to cell differentiation, proliferation, migration, apoptosis and senescence [26]. In particular, a large number of studies in recent years have illuminated the potential applications of lncRNAs as biomarkers or therapeutic targets in cancer progression and treatment tolerance [27–29]. In the previous study, we reported that overexpressed LINC00691 in GC tissues and serum was a potential biomarker for GC, which was correlated with tumor size, depth of invasion, lymph node metastasis, venous invasion, perineural invasion, and poor prognosis of patients [10]. We reported that LINC00691 promoted the proliferation and invasion of GC cells by activating Lin28 transcription and facilitating EGF expression through the JAK2/STAT3 signalling pathway. Other related studies reported that LINC00691 was abnormally expressed in non-small cell lung cancer, lung adenocarcinoma, osteosarcoma, and papillary thyroid cancer [30–33]. However, the functions of exosomal LINC00691 in GC remain unknown. In this study, we reported that LINC00691 expression in serum exosomes of GC patients was significantly higher than that of healthy volunteers and patients with gastric benign disease and it was associated with tumor size, lymph node metastasis, vascular invasion and perineural invasion of patients. The ROC curve showed that the sensitivity and specificity of diagnosis were: 0.85 and 0.71 respectively. However, limitations of using exosomal LINC00691 as a diagnosis biomarker for GC also exists. This result was based on a small local population, and the sample number of healthy volunteers in the control group was also small. More precise studies are needed in a larger population.

Increasing number of reports have indicated that tumor cell-derived exosomes play a prominent educational role in the tumor microenvironment [34]. Recent studies reported exosomes used as special drug delivery systems for targeted therapy reprogramming tumor microenvironment [35]. As important components of the microenvironment, fibroblasts may be involved in the activation of early oncogenic signals and regulate tumor progression after activation [36]. CAFs, considered as a set of activated fibroblasts produce extracellular matrix components such as cytokines, growth factors, and chemokines to support the growth and invasion of cancer cells [37]. CAFs are usually identified by detecting markers such as α-SMA, Vimentin, and fibroblast activation protein alpha (FAP) [38, 39]. We isolated CAFs and NFs from GC tissues and paired healthy tissues, and found that the expression levels of α-SMA and Vimentin were significantly higher in CAFs than that in NFs. After treatment with GC cell culture supernatant or exosomes, the expression of α-SMA and Vimentin in fibroblasts was significantly increased, which proved that fibroblasts were activated. Then the activation of fibroblasts was inhibited as exosomes in the supernatant of GC cells were deprived. Our results are consistent with the study reported [40, 41].

It was reported that conditioned media of CAFs promoted cancer calls to gain a more proliferative phenotype [42]. We threated GC cells with the culture supernatant of activated fibroblasts induced by exosomes and found that not only the proliferation of GC cells was enhanced, but also the migration and invasion were boosted. Radiotherapy to CAFs induced insulin-like growth factor-1 (IGF1) and stimulated survival of cancer cells, which was restrained by IGF1 receptor (IGF1R) neutralization [43]. This may be a potential therapeutic strategy targeting key soluble factors secreted by fibroblasts. LncRNA H19 was overexpressed in tumor tissues and facilitated the stemness and chemoresistance of colorectal cancer (CRC) through the regulation of CAFs [44]. We manifested that CAFs as well as activated fibroblasts induced by exosomes derived from GC cell culture supernatant expressed a higher level of LINC00691. Sh-RNAs targeted LINC00691 inhibited the effects of GC cell culture supernatant on fibroblasts. This study manifested that exosomes of GC cells promoted normal fibroblasts to gain more activated phenotypes similar to CAFs depending on LINC00691 partly at least. However, there are still more details about the underlying molecular mechanisms of exosomes-rich GC cell culture supernatant on normal fibroblasts needed clarified. Moreover, inhibitor of the JAK2/STAT3 signaling pathway ruxolitinib also suppressed the activation of normal fibroblasts. Biffi et al. reported that JAK/STAT activation was a dominant factor that promoted an inflammatory CAF state in pancreatic ductal adenocarcinoma (PDAC) [45]. Ruxolitinib is currently clinically applied for the treatment of patients with intermediate or high-risk myelofibrosis including primary myelofibrosis, post-polycythemia vera myelofibrosis and post-essential thrombocythemia myelofibrosis [46, 47]. In recent years, some studies have indicated that ruxolitinib may have potential applications in targeted therapy of other diseases, such as renal interstitial fibrosis [48], graft-versus-host disease [49] and severe coronavirus disease 2019 (COVID-19) [50].

Mainly, CAFs in GC were identified into two subsets, inflammatory CAFs (iCAFs) and extracellular matrix CAFs (eCAFs) by performing single-cell RNA sequencing [51]. Another study revealed myofibroblastic CAF (myCAF) and inflammatory CAF (iCAF) when tumors metastasized to the liver [52]. Although different CAF subtypes and classification are illustrated to have different biological functions, they are usually manifested to coordinate the tumor microenvironment to promote cancer progression [38]. In this study, we did not identify the subtype or classification of fibroblasts activated by exosomes in GC cell culture supernatants, which would be further explored in subsequent studies. Our study revealed that exosomal LINC00691 promoted NFs to obtain activated phenotype like CAFs through JAK/STAT signaling pathways. This study uncovered new therapeutic application in GC. However, there are more efforts needed to be put into practice before the clinical therapeutic application of ruxolitinib in GC. We need to verify the role of ruxolitinib in GC therapy in vivo in subsequent studies. Still there is no any clinical study having indicated that ruxolitinib benefits GC patients with a longer survival time.

Overall, our study indicated that serum exosomal LINC00691 was a potential diagnosis biomarker for GC. Exosomes derived from GC cell culture supernatant stimulated the activation of normal fibroblasts to gain a CAF-like phenotype, which performed a higher level of α-SMA and Vimentin and promoted the proliferation and invasion of GC cells.

### Supplementary Information

**Additional file 1: Table S1.** Clinicopathological features of gastric cancer patients. Alix and CD63 for Fig.3b. CD9 for Fig.3b. CD81 for Fig.3b. 

## Data Availability

All data generated or analyzed during this study are included in this published article (and its supplementary information files).

## Abbreviation

GC: Gastric cancer
NFs: Normal fibroblasts
CAFs: cancer-associated fibroblasts
JAK2/STAT3: Janus kinase 2 /signal transducer and activator of transcription 3
