# Supplementary material for: Exosomes-mediated transfer of LINC00691 regulates the formation of CAFs and promotes the progression of gastric cancer
Source: BMC Cancer. 2023 Oct 2;23:928. doi: 10.1186/s12885-023-11373-5 (PMC10544540; doi:10.1186/s12885-023-11373-5)

 Supplementary Information

Supplementary table 1 clinicopathological features of gastric cancer patients

| NO. | age | Histopathological typing (1-adenocarcinoma,2-Signet-ring cell carcinoma,3-Others) | Tumor size(cm, 1- ＜3,2- ≥3) | Tumor location(1-Cardia,2-Antrum ,3-Body,4-Others) | Lymphatic metastasis (1-Absent,2-Present) | Venous invasion(1-Absent,2-Present) | Perineural invasion (1-Absent,2-Present) | Invasion depth(1-T0,2-T1) | Differentiation(1-Poor,2-Moderate) | Gender(1-Male,2-Female) | expression of ex-LINC00691(-ddct) |
| --- | --- | --- | --- | --- | --- | --- | --- | --- | --- | --- | --- |
| 554690 | 79 | 3 | 1 | 1 | 1 | 1 | 1 | 1 | 1 | 1 | 0.003 |
| 555869 | 48 | 1 | 1 | 2 | 2 | 2 | 2 | 2 | 1 | 2 | 1.128 |
| 555349 | 77 | 1 | 1 | 2 | 1 | 1 | 1 | 1 | 1 | 1 | 0.933 |
| 556138 | 54 | 1 | 2 | 1 | 2 | 2 | 2 | 1 | 1 | 1 | 2.765 |
| 555916 | 69 | 1 | 1 | 1 | 1 | 1 | 1 | 1 | 1 | 1 | 1.042 |
| 557006 | 54 | 2 | 1 | 2 | 2 | 1 | 1 | 2 | 1 | 1 | -3.923 |
| 551681 | 63 | 1 | 1 | 2 | 1 | 1 | 1 | 1 | 1 | 1 | -0.884 |
| 557768 | 60 | 1 | 1 | 2 | 1 | 2 | 1 | 2 | 1 | 1 | -1.604 |
| 556718 | 70 | 1 | 1 | 1 | 2 | 2 | 1 | 2 | 1 | 2 | 1.584 |
| 558230 | 66 | 1 | 1 | 3 | 2 | 2 | 2 | 2 | 1 | 1 | 0.134 |
| 558188 | 72 | 1 | 1 | 1 | 1 | 1 | 1 | 2 | 1 | 1 | -0.891 |
| 558522 | 69 | 1 | 2 | 3 | 1 | 2 | 1 | 1 | 1 | 1 | 2.409 |
| 559044 | 69 | 3 | 2 | 4 | 2 | 2 | 2 | 2 | 1 | 1 | 2.776 |
| 558686 | 79 | 1 | 1 | 3 | 2 | 1 | 1 | 2 | 1 | 1 | -0.860 |
| 558958 | 66 | 1 | 2 | 1 | 2 | 2 | 2 | 2 | 2 | 1 | 3.156 |
| 559125 | 78 | 2 | 1 | 1 | 2 | 2 | 1 | 2 | 2 | 2 | -0.105 |
| 559076 | 68 | 1 | 1 | 2 | 2 | 1 | 1 | 2 | 1 | 1 | 2.757 |
| 559207 | 77 | 1 | 1 | 2 | 1 | 1 | 1 | 1 | 2 | 1 | 1.447 |
| 559365 | 67 | 1 | 2 | 1 | 1 | 1 | 1 | 1 | 1 | 1 | 3.731 |
| 559458 | 69 | 1 | 1 | 1 | 1 | 1 | 1 | 1 | 2 | 2 | -16.791 |
| 559593 | 51 | 1 | 1 | 1 | 1 | 1 | 1 | 1 | 2 | 2 | 2.362 |
| 558171 | 62 | 1 | 2 | 4 | 1 | 1 | 1 | 2 | 1 | 1 | 0.224 |
| 559968 | 83 | 1 | 1 | 1 | 1 | 1 | 1 | 1 | 1 | 1 | -2.062 |

| Supplementary table 1 continued clinicopathological features of gastric cancer patients | | | | | | | | | | | |
| --- | --- | --- | --- | --- | --- | --- | --- | --- | --- | --- | --- |
| NO. | age | Histopathological typing (1-adenocarcinoma,2-Signet-ring cell carcinoma,3-Others) | Tumor size(cm, 1- ＜3,2- ≥3) | Tumor location(1-Cardia,2-Antrum ,3-Body,4-Others) | Lymphatic metastasis (1-Absent,2-Present) | Venous invasion(1-Absent,2-Present) | Perineural invasion (1-Absent,2-Present) | Invasion depth(1-T0,2-T1) | Differentiation(1-Poor,2-Moderate) | Gender(1-Male,2-Female) | expression of ex-LINC00691(-ddct) |
| 560027 | 47 | 1 | 1 | 1 | 1 | 1 | 1 | 1 | 2 | 2 | 1.878 |
| 559904 | 79 | 1 | 2 | 2 | 2 | 2 | 2 | 2 | 1 | 1 | 4.208 |
| 559903 | 64 | 1 | 1 | 4 | 2 | 2 | 1 | 2 | 1 | 2 | 0.506 |
| 560473 | 76 | 1 | 2 | 2 | 2 | 1 | 2 | 2 | 1 | 2 | -1.996 |
| 560645 | 77 | 1 | 2 | 3 | 2 | 2 | 2 | 2 | 1 | 1 | -15.880 |
| 559339 | 66 | 3 | 1 | 4 | 2 | 1 | 1 | 1 | 2 | 1 | -0.724 |
| 561491 | 75 | 1 | 2 | 3 | 2 | 2 | 2 | 2 | 1 | 1 | 2.329 |
| 561448 | 68 | 1 | 2 | 4 | 2 | 2 | 2 | 2 | 1 | 1 | 0.195 |
| 561742 | 62 | 1 | 2 | 1 | 2 | 2 | 2 | 2 | 1 | 1 | 5.913 |
| 561362 | 58 | 1 | 2 | 3 | 1 | 1 | 1 | 1 | 1 | 1 | 6.788 |
| 561604 | 82 | 1 | 1 | 1 | 1 | 1 | 1 | 1 | 1 | 1 | 0.881 |
| 561779 | 56 | 1 | 1 | 2 | 1 | 1 | 1 | 1 | 1 | 2 | -8.564 |
| 561525 | 83 | 1 | 2 | 2 | 2 | 2 | 2 | 2 | 1 | 2 | 4.624 |
| 561754 | 69 | 1 | 2 | 1 | 2 | 2 | 2 | 2 | 1 | 1 | 4.757 |
| 562036 | 81 | 1 | 2 | 2 | 2 | 1 | 1 | 1 | 1 | 2 | 2.764 |
| 562605 | 54 | 2 | 1 | 2 | 1 | 1 | 1 | 2 | 2 | 2 | 0.134 |
| 562579 | 52 | 1 | 1 | 2 | 1 | 1 | 1 | 1 | 1 | 1 | -0.072 |
| 562708 | 52 | 1 | 2 | 3 | 2 | 1 | 2 | 2 | 1 | 1 | 4.738 |
| 562874 | 46 | 1 | 2 | 3 | 2 | 1 | 1 | 2 | 1 | 2 | 3.654 |
| 563011 | 77 | 1 | 2 | 3 | 2 | 2 | 1 | 2 | 1 | 1 | 7.439 |
| 563097 | 60 | 1 | 1 | 3 | 2 | 2 | 1 | 2 | 1 | 1 | -0.035 |
| 563172 | 53 | 1 | 1 | 1 | 2 | 1 | 1 | 1 | 1 | 1 | -1.902 |
| 563603 | 64 | 1 | 2 | 4 | 2 | 2 | 2 | 2 | 1 | 1 | 2.747 |

|  | | | | | | | | | | | | | | | | | | | |
| --- | --- | --- | --- | --- | --- | --- | --- | --- | --- | --- | --- | --- | --- | --- | --- | --- | --- | --- | --- |
| Supplementary table 1 clinicopathological features of gastric cancer patients | | | | | | | | | | | | | | | | | | | |
| NO. | age | | Histopathological typing (1-adenocarcinoma,2-Signet-ring cell carcinoma,3-Others) | | Tumor size(cm, 1- ＜3,2- ≥3) | | Tumor location(1-Cardia,2-Antrum ,3-Body,4-Others) | | Lymphatic metastasis (1-Absent,2-Present) | | Venous invasion(1-Absent,2-Present) | | Perineural invasion (1-Absent,2-Present) | | Invasion depth(1-T0,2-T1) | | Differentiation(1-Poor,2-Moderate) | Gender(1-Male,2-Female) | expression of ex-LINC00691(-ddct) |
| 563809 | 51 | 1 | | 1 | | 3 | | 1 | | 1 | | 1 | | 1 | | 1 | | 1 | -0.904 |
| 563667 | 72 | 1 | | 2 | | 4 | | 1 | | 1 | | 1 | | 1 | | 2 | | 1 | 2.127 |
| 301961105 | 67 | 1 | | 1 | | 1 | | 1 | | 1 | | 1 | | 1 | | 2 | | 1 | -0.955 |
| 564196 | 64 | 2 | | 2 | | 1 | | 2 | | 1 | | 2 | | 2 | | 1 | | 1 | -0.803 |
| 564438 | 74 | 1 | | 1 | | 4 | | 1 | | 1 | | 1 | | 1 | | 1 | | 2 | -0.155 |
| 564411 | 74 | 1 | | 1 | | 1 | | 2 | | 2 | | 1 | | 1 | | 1 | | 2 | 0.733 |
| 564891 | 78 | 1 | | 1 | | 3 | | 1 | | 1 | | 1 | | 1 | | 2 | | 1 | -0.509 |
| 564953 | 69 | 1 | | 2 | | 2 | | 2 | | 2 | | 2 | | 2 | | 2 | | 1 | 2.829 |
| 565203 | 71 | 1 | | 2 | | 1 | | 2 | | 1 | | 1 | | 1 | | 1 | | 1 | 1.979 |
| 565594 | 55 | 1 | | 2 | | 2 | | 2 | | 2 | | 1 | | 2 | | 1 | | 2 | 2.844 |
| 565375 | 69 | 1 | | 1 | | 2 | | 1 | | 1 | | 1 | | 1 | | 1 | | 1 | -1.746 |
| 565928 | 71 | 1 | | 1 | | 3 | | 1 | | 2 | | 2 | | 2 | | 1 | | 1 | 3.129 |
| 566115 | 80 | 1 | | 2 | | 2 | | 1 | | 1 | | 1 | | 2 | | 1 | | 1 | -0.419 |
| 566617 | 48 | 1 | | 1 | | 2 | | 1 | | 1 | | 2 | | 2 | | 1 | | 1 | 1.132 |
| 566327 | 60 | 1 | | 2 | | 3 | | 2 | | 2 | | 2 | | 2 | | 2 | | 1 | 1.530 |
| 566240 | 59 | 2 | | 1 | | 3 | | 1 | | 1 | | 1 | | 1 | | 1 | | 2 | 0.099 |
| 307340713 | 63 | 1 | | 1 | | 3 | | 1 | | 1 | | 1 | | 1 | | 2 | | 2 | 3.211 |
| 566778 | 65 | 1 | | 1 | | 3 | | 1 | | 1 | | 1 | | 1 | | 2 | | 1 | -1.000 |
| 566719 | 63 | 1 | | 1 | | 2 | | 1 | | 1 | | 1 | | 1 | | 2 | | 1 | 0.258 |
| 566954 | 56 | 1 | | 1 | | 4 | | 2 | | 2 | | 2 | | 2 | | 1 | | 1 | 5.687 |
| 567210 | 57 | 1 | | 1 | | 2 | | 1 | | 1 | | 1 | | 2 | | 1 | | 1 | 0.866 |
| 567207 | 56 | 2 | | 2 | | 3 | | 1 | | 1 | | 1 | | 2 | | 2 | | 2 | 12.652 |
| 566983 | 66 | 1 | | 1 | | 1 | | 2 | | 2 | | 2 | | 2 | | 2 | | 1 | 0.286 |

| Supplementary table 1 clinicopathological features of gastric cancer patients | | | | | | | | | | | | | | | | | | | | |
| --- | --- | --- | --- | --- | --- | --- | --- | --- | --- | --- | --- | --- | --- | --- | --- | --- | --- | --- | --- | --- |
| NO. | age | | Histopathological typing (1-adenocarcinoma,2-Signet-ring cell carcinoma,3-Others) | | Tumor size(cm, 1- ＜3,2- ≥3) | | Tumor location(1-Cardia,2-Antrum ,3-Body,4-Others) | | Lymphatic metastasis (1-Absent,2-Present) | | Venous invasion(1-Absent,2-Present) | | Perineural invasion (1-Absent,2-Present) | | Invasion depth(1-T0,2-T1) | | Differentiation(1-Poor,2-Moderate) | Gender(1-Male,2-Female) | | expression of ex-LINC00691(-ddct) |
| 567815 | 67 | 1 | | 1 | | 2 | | 1 | | 1 | | 1 | | 1 | | 1 | | | 1 | 1.040 |
| 567560 | 81 | 1 | | 2 | | 1 | | 2 | | 1 | | 2 | | 2 | | 1 | | | 2 | 1.118 |
| 567780 | 56 | 1 | | 1 | | 1 | | 2 | | 1 | | 1 | | 1 | | 1 | | | 1 | 2.279 |
| 567568 | 79 | 1 | | 2 | | 3 | | 2 | | 2 | | 2 | | 2 | | 1 | | | 1 | 3.970 |
| SA22754949 | 53 | 1 | | 1 | | 1 | | 1 | | 1 | | 1 | | 1 | | 2 | | | 2 | 0.107 |
| 568066 | 71 | 1 | | 2 | | 1 | | 2 | | 2 | | 2 | | 2 | | 1 | | | 1 | 1.039 |
| 568310 | 57 | 3 | | 1 | | 1 | | 1 | | 1 | | 1 | | 2 | | 2 | | | 1 | 0.501 |
| 568210 | 59 | 1 | | 2 | | 1 | | 2 | | 2 | | 2 | | 2 | | 1 | | | 1 | 2.501 |
| 569070 | 55 | 1 | | 1 | | 1 | | 1 | | 1 | | 1 | | 2 | | 1 | | | 1 | 0.254 |
| 569130 | 70 | 1 | | 1 | | 1 | | 1 | | 1 | | 1 | | 1 | | 1 | | | 1 | -0.393 |
| 569475 | 61 | 1 | | 2 | | 3 | | 2 | | 1 | | 1 | | 2 | | 1 | | | 1 | 4.580 |
| 568310 | 57 | 3 | | 2 | | 1 | | 2 | | 1 | | 1 | | 2 | | 2 | | | 1 | 1.368 |
| 570083 569702 | 77 | 1 | | 1 | | 1 | | 1 | | 1 | | 1 | | 1 | | 2 | | | 1 | 0.344 |
| 569756 | 79 | 1 | | 2 | | 3 | | 2 | | 2 | | 2 | | 2 | | 1 | | | 1 | 0.795 |
| 570012 | 56 | 1 | | 1 | | 1 | | 1 | | 1 | | 1 | | 1 | | 1 | | | 1 | -0.280 |
| 569430 | 60 | 1 | | 1 | | 3 | | 2 | | 2 | | 1 | | 2 | | 1 | | | 1 | 1.552 |
| 570007 | 71 | 1 | | 1 | | 3 | | 1 | | 1 | | 1 | | 2 | | 1 | | | 1 | -2.965 |
| 570517 | 61 | 1 | | 1 | | 1 | | 1 | | 1 | | 1 | | 1 | | 2 | | | 2 | 0.065 |
| 570376 | 77 | 1 | | 2 | | 3 | | 2 | | 2 | | 2 | | 2 | | 1 | | | 1 | 2.080 |

|  | | | | | | | | | | | | | | | | | | | | |
| --- | --- | --- | --- | --- | --- | --- | --- | --- | --- | --- | --- | --- | --- | --- | --- | --- | --- | --- | --- | --- |
| Supplementary table 1 clinicopathological features of gastric cancer patients | | | | | | | | | | | | | | | | | | | | |
| NO. | age | | Histopathological typing (1-adenocarcinoma,2-Signet-ring cell carcinoma,3-Others) | | Tumor size(cm, 1- ＜3,2- ≥3) | | Tumor location(1-Cardia,2-Antrum ,3-Body,4-Others) | | Lymphatic metastasis (1-Absent,2-Present) | | Venous invasion(1-Absent,2-Present) | | Perineural invasion (1-Absent,2-Present) | | Invasion depth(1-T0,2-T1) | | Differentiation(1-Poor,2-Moderate) | Gender(1-Male,2-Female) | | expression of ex-LINC00691(-ddct) |
| 307177320 | 52 | 1 | | 1 | | 1 | | 1 | | 1 | | 1 | | 1 | | 2 | | | 2 | 0.405 |
| 571276 | 76 | 2 | | 1 | | 1 | | 1 | | 1 | | 1 | | 1 | | 1 | | | 1 | -0.189 |
| 571704 | 73 | 2 | | 1 | | 1 | | 1 | | 1 | | 1 | | 2 | | 1 | | | 2 | 0.148 |
| 572408 | 63 | 3 | | 1 | | 3 | | 1 | | 1 | | 1 | | 1 | | 2 | | | 2 | 5.315 |
| Y0502510987 | 59 | 1 | | 1 | | 1 | | 1 | | 1 | | 1 | | 1 | | 2 | | | 1 | 0.785 |

Alix and CD63 for Fig.3b


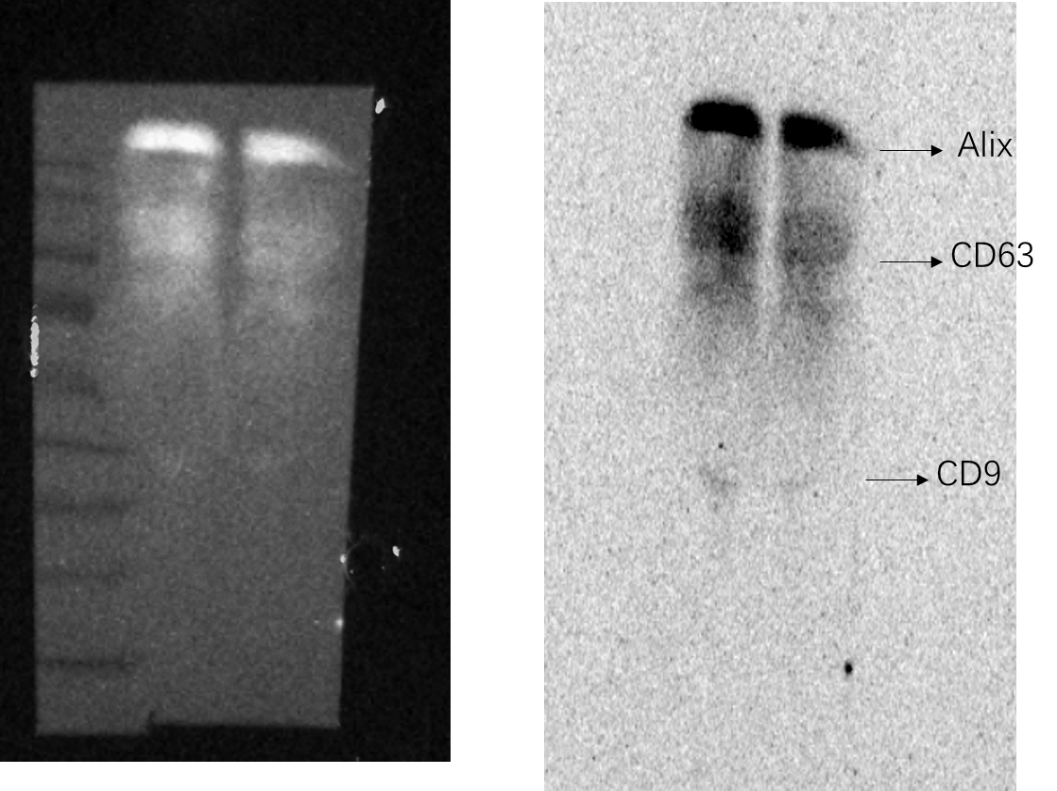


CD9 for Fig.3b


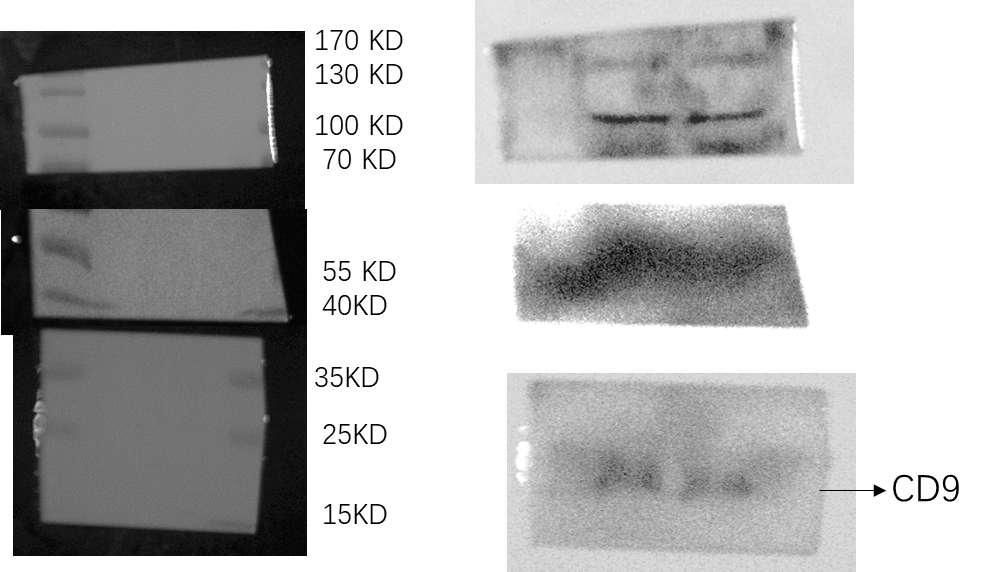


CD81 for Fig.3b


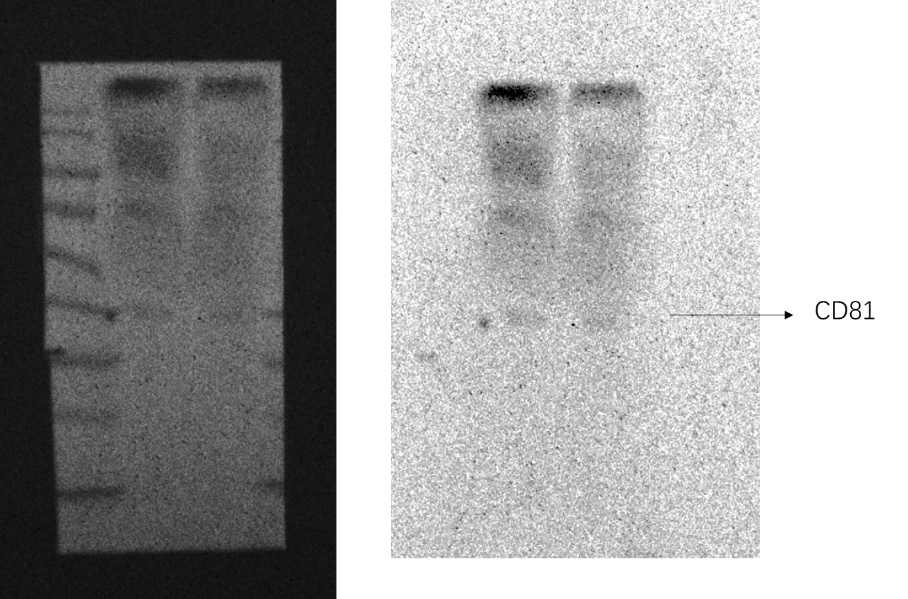

Supplement: Supplementary file 1 — Additional file 1: Table S1. Clinicopathological features of gastric cancer patients. Alix and CD63 for Fig.3b. CD9 for Fig.3b. CD81 for Fig.3b. [file 12885_2023_11373_MOESM1_ESM.docx]
